# Dieback and Replacement of Riparian Trees May Impact Stream Ecosystem Functioning

**DOI:** 10.1007/s00248-024-02343-w

**Published:** 2024-01-16

**Authors:** Alberto Alonso, Luz Boyero, Alejandro Solla, Verónica Ferreira

**Affiliations:** 1 Department of Plant Biology and Ecology, Faculty of Science and Technology, University of the Basque Country (UPV/EHU), Leioa, Spain; 2 Basque Foundation for Science, IKERBASQUE, Bilbao, Spain; 3 Faculty of Forestry, Institute for Dehesa Research (INDEHESA), Universidad de Extremadura, Avenida Virgen del Puerto 2, 10600 Plasencia, Spain; 4 Marine and Environmental Sciences Centre (MARE), Aquatic Research Network (ARNET), Department of Life Sciences, University of Coimbra, Calçada Martim de Freitas, 3000-456 Coimbra, Portugal

**Keywords:** Alder, *Alnus lusitanica*, Black locust, *Robinia pseudoacacia*, *Phytophthora* ×*alni*, Leaf litter decomposition

## Abstract

Alders are nitrogen (N)-fixing riparian trees that promote leaf litter decomposition in streams through their high-nutrient leaf litter inputs. While alders are widespread across Europe, their populations are at risk due to infection by the oomycete *Phytophthora* ×*alni*, which causes alder dieback. Moreover, alder death opens a space for the establishment of an aggressive N-fixing invasive species, the black locust (*Robinia pseudoacacia*). Shifts from riparian vegetation containing healthy to infected alder and, eventually, alder loss and replacement with black locust may alter the key process of leaf litter decomposition and associated microbial decomposer assemblages. We examined this question in a microcosm experiment comparing three types of leaf litter mixtures: one representing an original riparian forest composed of healthy alder (*Alnus lusitanica*), ash (*Fraxinus angustifolia*), and poplar (*Populus nigra*); one with the same species composition where alder had been infected by *P.* ×*alni*; and one where alder had been replaced with black locust. The experiment lasted six weeks, and every two weeks, microbially driven decomposition, fungal biomass, reproduction, and assemblage structure were measured. Decomposition was highest in mixtures with infected alder and lowest in mixtures with black locust, reflecting differences in leaf nutrient concentrations. Mixtures with alder showed distinct fungal assemblages and higher sporulation rates than mixtures with black locust. Our results indicate that alder loss and its replacement with black locust may alter key stream ecosystem processes and assemblages, with important changes already occurring during alder infection. This highlights the importance of maintaining heathy riparian forests to preserve proper stream ecosystem functioning.

**Supplementary Information:**

The online version contains supplementary material available at 10.1007/s00248-024-02343-w.

## Introduction

The functioning of stream ecosystems can be highly influenced by changes in the riparian forest [1, 2]. This applies especially to headwater streams, where the major basal resource is the allochthonous organic material provided by the riparian vegetation [3], mainly in the form of leaf litter [4–6]. Given that different tree species produce leaf litter with different traits [7–9], species composition of the riparian forest can determine how this leaf litter is processed in the stream and therefore partially regulate its decomposition rates, the transfer of energy and nutrients between ecosystem compartments [10], and the characteristics of stream assemblages involved in these processes [11–13]. In consequence, changes in riparian species composition, which can occur through species loss (e.g., caused by pathogenic diseases or the long term result of biological invasions [4, 14]), species gain or species replacement (e.g., at initial stages of biological invasions [15]), are expected to be highly relevant to predict changes in stream ecosystem functioning.

Alders (*Alnus* spp.) are key riparian trees across Europe and often the only nitrogen (N)-fixing native tree species present [16]. Their leaves are soft and high in N concentration [16], which makes them highly attractive to microbial decomposers and detritivores, hence allowing rapid leaf decomposition in streams [17]. However, alders are suffering widespread mortality due to the *Alnus*-specific oomycete species complex *Phytophthora alni* [18–21]. In Spain and Portugal, alder mortality has been observed since the mid-2000s and *P*. ×*alni* (Brasier & S.A. Kirk) Husson, Ioos and Marçais was first isolated in 2009 and 2010, respectively [22, 23]. In the northern regions, disease severity has been high, leading to the disappearance of most of the trees in Asturias and northern Galicia. At the southern limit of *P*. ×*alni* distribution, occurring in Jerte river (Plasencia, Spain) [24] and Ceira river (Arouce, Portugal) [25], disease severity is lower than in the north. Tree infection is produced by zoospores in the roots or in the trunk during floods, leading to root rot, collar rot, small-size, sparse and often chlorotic foliage, crown dieback, and tree mortality. Mortality rates reach almost 100%, with young trees usually dying in few months and old trees dying in years while losing vitality progressively [18, 19]. Leaf litter of *Alnus lusitanica* Vít, Douda and Mandák infected trees has higher nutrient concentration than that from healthy trees due to reduced nutrient resorption before senescence, leading to fast decomposition of leaf litter [24].

When key tree species disappear from forests, exotic species can readily occupy their niche [26]. Riparian forests are particularly prone to invasions because streams act as corridors favoring propagule transport, cause natural disturbances (i.e., floods) that open canopy gaps, and alter micro-climatic conditions thus favoring plant establishment [27–29]. Occurrence of invasion is higher in riparian areas affected by human activities (e.g., forestry, agriculture or urbanization), where propagule pressure is higher and the colonization of shade sensitive invasive species is favored by large forest gaps [15, 30]. A likely species to replace alder after its disappearance is the black locust (*Robinia pseudoacacia* L.), an N-fixing tree species native to North America that has become a major invasive species in European riparian forests due to its fast growth, high resistance to disturbance, and low nutrient requirements [26, 31, 32]. Despite the high N concentration of black locust leaf litter, it decomposes more slowly than leaf litter of many native tree species (e.g., *Salix atrocinerea* Brot., *Fraxinus angustifolia* Vahl. or *Populus alba* L.), possibly because its high concentration of secondary compounds and lignin negatively affects microbial colonization and macroinvertebrate assemblages [33–35].

We explored whether alder infection by *P.* ×*alni* and subsequent alder replacement by black locust affects stream ecosystem functioning. Microbially-driven leaf litter decomposition and the characteristics of associated fungal assemblages were assessed. With this aim, we conducted a microcosm experiment using leaf litter and simulated the following scenarios: (1) a native riparian forest containing ash (*F. angustifolia*), poplar (*Populus nigra* L.), and healthy alder (*A. lusitanica*, previously *Alnus glutinosa* (L.) Gaertn.); (2) the same forest but with alder infected by *P.* ×*alni*, representing an early stage of an epidemic; and (3) the same forest but with alder replaced by black locust, representing a post-epidemic stage. To explore differences among native ash, native poplar, healthy and infected native alder, and invasive black locust, and facilitate the understanding of interactions in leaf mixtures, including diversity effects on decomposition and fungal biomass, monocultures of all leaf litter types were also assessed. Based on the main traits of different leaf litter types, we hypothesized the following:

(i) Scenario 2, with infected alder, would show higher leaf litter decomposition rate, fungal biomass, and fungal sporulation rate than scenario 1, with healthy alder, since leaf litter of infected alder is richer in nutrients and more labile [24], thus possibly enhancing decomposition and fungal activity in mixtures [12].
(ii) Scenario 3, with black locust, would show lower leaf litter decomposition rate, fungal biomass, and fungal sporulation rate than scenarios 1 and 2 (with healthy and infected alder, respectively), because of the higher lignin and polyphenol concentration in black locust leaf litter, which potentially slows down decomposition and fungal activity in mixtures [12], despite both black locust and alder having high concentration of nutrients [33, 34].
(iii) Fungal assemblages would be altered in scenarios 2 and 3 compared with scenario 1, due to strong substrate preferences of fungal microorganisms [11], the change being higher under scenario 3 than under scenario 2 because fungi would be more affected by species composition change [12] than by alder infection [24].

## Materials and Methods

### Leaf Litter Collection

The four plant species used in the experiment (ash, poplar, alder, and black locust) are broadleaf deciduous trees that range widely in leaf litter traits (Table 1). Leaves of poplar, ash and black locust were collected immediately after natural abscission from the floor in the floodplain of the Mondego river (Coimbra, central Portugal), at Choupalinho (40°12′4.7″N, 8°25′42.9″W, in autumn 2020), Parque Verde (40°12′3.2″N, 8°25′29.7″W, in autumn 2022), and Mata National do Choupal (40°13′4.3″N, 8°26′28.4″W, in autumn 2022), respectively. Senescent alder leaves were gently detached from healthy trees (≤ 5% crown transparency estimated visually) and trees infected by *P.* ×*alni* (≥ 60% crown transparency) located in the floodplain of the Jerte river (Plasencia, Spain; 40°1′51.2″N, 6°4′46.0″W, in autumn 2017) [24]. Isolations of *P*. ×*alni* from bark samples, including the cambium [20], confirmed infection of the trees, whereas no pathogen was isolated from bark samples of healthy trees. Leaf litter was air-dried at room temperature in the laboratory and stored in the dark until needed.

**Table 1:** Initial traits of the different leaf litter types used in the study (mean ± s.e.; *n *= 3): carbon, nitrogen, phosphorus, polyphenol, and lignin concentrations, elemental molar ratios, and leaf toughness

Leaf litter traits	*Fraxinus angustifolia*	*Populus nigra*	*Alnus lusitanica* healthy	*Alnus lusitanica* infected	*Robinia pseudoacacia*
Carbon (C; % DM)***	44.16 ± 0.10^c^	42.00 ± 0.20^e^	47.62 ± 0.09^a^	46.48 ± 0.19^b^	43.44 ± 0.21^d^
Nitrogen (N; % DM)***	0.75 ± 0.02^d^	0.87 ± 0.02^c^	3.80 ± 0.73^a^	2.52 ± 0.08^a^	1.33 ± 0.01^b^
Phosphorus (P; % DM)***	0.17 ± 0.01^a^	0.14 ± 0.01^b^	0.06 ± 0.00^cd^	0.14 ± 0.05^abc^	0.04 ± 0.01^d^
Polyphenol (% DM)**	9.62 ± 0.28^b^	16.30 ± 4.13^ab^	8.50 ± 0.83^b^	9.83 ± 0.33^b^	11.86 ± 0.43^a^
Lignin (% DM)***	17.99 ± 0.35^c^	35.88 ± 0.11^a^	37.06 ± 1.99^ab^	36.74 ± 1.56^ab^	34.06 ±0.43^b^
C:N***	59.25 ± 1.92^a^	48.52 ± 1.30^b^	13.69 ± 3.00^d^	18.49 ± 0.53^d^	32.70 ± 0.26^c^
C:P***	253.69 ± 8.04^c^	304.56 ± 14.39^b^	845.74 ± 54.69^a^	422.71 ± 114.52^bc^	1092.26 ± 165.87^a^
N:P***	4.29 ± 0.22^d^	6.28 ± 0.23^c^	65.85 ± 9.35^a^	23.22 ± 6.66^b^	33.38 ± 5.00^b^
Toughness (kPa)***	801 ± 96^ab^	898 ± 77^a^	609 ± 52^b^	551 ± 111^abc^	382 ± 9^c^

For each trait, different letters indicate significant differences among leaf litter types, analyzed with linear models

*p < 0.05; **p < 0.01; ***p < 0.001

### Leaf Litter Characterization

Initial characterization was performed using three replicates per leaf litter type. Air-dried leaf litter was milled into fine powder (< 0.5 mm; Retsch MM 400, Haan, Germany), oven-dried at 60°C for 48 h, and used for chemical determinations. Carbon (C) and N concentrations were assessed by isotope ratio mass spectrophotometry (IRMS Thermo Delta V advantage with a Flash EA-1112 series; Thermo Fisher Scientific Inc., Waltham, USA). Phosphorous (P) concentration was assessed by the ascorbic acid method after basic digestion with sodium persulfate and sodium hydroxide [36]. Total polyphenol concentration was obtained by the Folin-Ciocalteu method [36] and lignin concentration by the Goering-van Soest method [37]. Concentrations were expressed as % dry mass (DM). Initial litter toughness (kPa) was estimated with a penetrometer (rod diameter 1.55 mm) after one hour of soaking in distilled water [36].

### Experimental Procedure

The experimental design included eight treatments: five monocultures (ash, poplar, healthy and infected alder, and black locust) and three mixtures, called scenarios, with three species each: ash, poplar, and healthy alder (scenario 1); ash, poplar, and infected alder (scenario 2); and ash, poplar, and black locust (scenario 3). Each treatment included three replicates collected at each of four sampling dates; thus, the experiment comprised 96 microcosms in total. Before the beginning of the experiment, leaf litter was moistened with distilled water, and 6-mm diameter discs were cut avoiding central veins (except for the narrow ash leaves, where discs included the vein in the center). Discs were air-dried at room temperature for 72 h and weighed (± 0.1 mg) in groups of 12, either of the same species (monoculture) or 4 discs per species (mixtures, with each species weighed individually). Discs were then distributed in 100-mL Erlenmeyer flasks (microcosms), which were assembled on an orbital shaker (100 rpm; GFL 3017, ProfiLab24 GmbH, Berlin, Germany) and kept under controlled conditions (21°C and 12 h light:12 h dark photoperiod).

For the first seven days, microcosms were supplied daily with 40 mL of a microbial inoculum <24 h old, to allow for leaching and microbial colonization of leaf litter discs. The inoculum was prepared by incubating a diverse mixture of leaf litter at different decomposition degrees in a glass jar with 4 L of filtered (100 μm) stream water and aeration, kept at 21°C, with water renewal every 24 h. The litter and water were collected in October 2022 from Candal stream (Lousã mountain, central Portugal; 40°4′44.7″N, 8°12′11.3″W), an oligotrophic stream with riparian vegetation at the sampling site dominated by European chestnut trees (*Castanea sativa* Mill.), and from where the tree species used in this study were absent [6]. A set of three microcosms per treatment (i.e., 24 microcosms) was sacrificed after the conditioning period (day 0) and processed as the experimental microcosms (see below), to obtain a correction factor to estimate initial, post-leaching, litter DM.

At day 0, all other experimental microcosms were supplied with 40 mL of filtered stream water (Candal stream), which was renewed every 3.5 days. Three replicates of each treatment were sacrificed at days 14, 28, and 42 to assess remaining leaf litter mass, fungal biomass, and conidial production. All litter discs were frozen at −20°C, lyophilized overnight (Lablyo Mini, Frozen in Time, North Yorkshire, UK), and weighed for determination of DM remaining, with species in mixtures weighed individually.

### Fungal Conidial Production

At each sampling date (days 14, 28, and 42), conidial suspensions from the sacrificed microcosms (40 mL) were poured into 50-mL centrifuge tubes, preserved with 2 mL of 37% formalin, adjusted to a volume of 45 mL with distilled water, and stored in the dark until processed. Samples were processed to determine sporulation rates and assemblage structure of aquatic hyphomycetes, a polyphyletic group of aquatic fungi assumed to be major microbial decomposers [38, 39]. Each conidial suspension received 100 μL of 0.5 % Triton X-100 and was homogenized with a magnetic stirrer. Then, 10 mL was filtered through nitro-cellulose filters (25-mm diameter, 5-μm pore size; Sartorius Stedim Biotech GmbH, Goettingen, Germany), and filters were stained with 0.05% cotton blue in 60% lactic acid. Conidia were identified and counted with a microscope (Leica, DM1000, Wetzlar, Germany) at ×200 magnification [36]. Sporulation rates were expressed as number of conidia mg^−1^ DM d^−1^, and species richness was expressed as number of species per sample.

### Fungal Biomass

At days 14 and 42, four discs from each species per microcosm (i.e., 4 discs in monocultures or the 4 discs of each species in mixtures) were used to determine fungal biomass from ergosterol concentration [36]. Discs were weighed to determine DM and ergosterol was extracted in 10 mL of alkaline methanol (8 g KOH/L) in a hot bath (80°C, 30 min), purified by solid phase extraction (Waters Sep-Pak Vac RC, 500 mg, Tc18 cartridges; Waters Corp., Milford, MA, USA), and quantified with high-performance liquid chromatography (Dionex DX-120; Sunnyvale, CA, USA) by measuring absorbance at 282 nm [36]. Ergosterol concentration was converted into fungal biomass assuming 5.5 μg ergosterol mg^−1^ fungal DM [40], and results were expressed as mg fungal DM.

### Data Analyses

The fraction of leaf litter DM remaining per species and mixture was calculated by dividing DM remaining by initial DM. Decomposition rates (*k*, day^−1^) were calculated for each species and mixture assuming an exponential decay model, through linear regression of the ln-transformed fraction of DM remaining over time, considering the intercept fixed at ln(1)=0. Net diversity, complementarity, and selection effects on decomposition were also calculated [41]; the net diversity effect was the difference between observed and expected leaf litter mass loss (LML, calculated as the difference between initial DM and DM remaining divided by initial DM), with expected fraction LML calculated as the mean of monoculture values taking into account the proportion of each species in the mixture; the complementarity effect was the average deviation from the expected fraction LML in a mixture multiplied by mean fraction LML in monocultures and the number of species in the mixture; and the selection effect was the covariance between fraction LML of species in monoculture and the average deviation from expected fraction LML of species in the mixture, multiplied by the number of species in the mixture. Net diversity, complementarity, and selection effects on fungal biomass was calculated in the same way using fungal biomass of each species in monocultures and mixtures.

Initial leaf litter traits were compared among leaf litter types (ash, poplar, healthy alder, infected alder, and black locust) with linear models and ANOVA (*gls* function of the “nlme” R package), with species as fixed factor. Effects of leaf litter type (five types) and scenario (1, 2 or 3) on different response variables were analyzed separately. To assess leaf litter decomposition, we used analysis of covariance (ANCOVA, *aov* function of the “stats” R package), with fraction DM remaining as a dependent variable, treatment (leaf litter type or scenario) as a categorical factor and time as a covariate. To assess fungal biomass, sporulation rate and species richness, and net, complementarity, and selection effects on decomposition and fungal biomass, we used linear models and ANOVA, with treatment (leaf litter type or scenario) and time as fixed factors. Significant differences between treatments (*α* = 0.05) were analyzed with Tukey’s tests, or Fisher’s LSD tests when Tukey’s tests did not identify differences among treatments [i..e., for species richness and net complementarity and selection effects; ghlt function of the “multcomp” R package; [42]]. Differences in fungal assemblages among treatments and sampling dates were explored with non-metric multidimensional scaling (NMDS) based on the Bray-Curtis similarity index applied to an abundance matrix (*metaMDS* function of the “vegan” R package), followed by permutational multivariate analysis of variance (PERMANOVA; *adonis* function of the “vegan” R package). An indicator value index (*multipatt* function of the “indicspecies” R package) was used to identify the most representative taxa of each assemblage. Normal distribution of residuals was assessed by the Shapiro-Wilk test, and data were log-transformed when non-normal distribution was detected. All analyses were performed in R software [43].

## Results

### Leaf Litter Traits

Initial traits varied with the type of leaf litter (Table 1, Table S1). Native species (ash, poplar, and healthy alder) showed a gradient, with N and lignin concentrations and C:P and N:P ratios being highest in healthy alder and lowest in ash, while P concentration and C:N showed the opposite pattern; C concentration was lowest in poplar and highest in healthy alder. Healthy and infected alder differed in C concentration and C:P and N:P ratios, which were higher in healthy alder. Black locust had higher polyphenol concentration than the other species except poplar; the lowest toughness, although not significantly different from infected alder; and higher lignin concentration than ash and lower than poplar. Black locust had lower N concentration than alder, but higher than ash and poplar; the lowest P concentration, but not significantly different from healthy alder; and the second lowest C concentration, after poplar (Table 1).

### Leaf Litter Decomposition

**Fig. 1:**
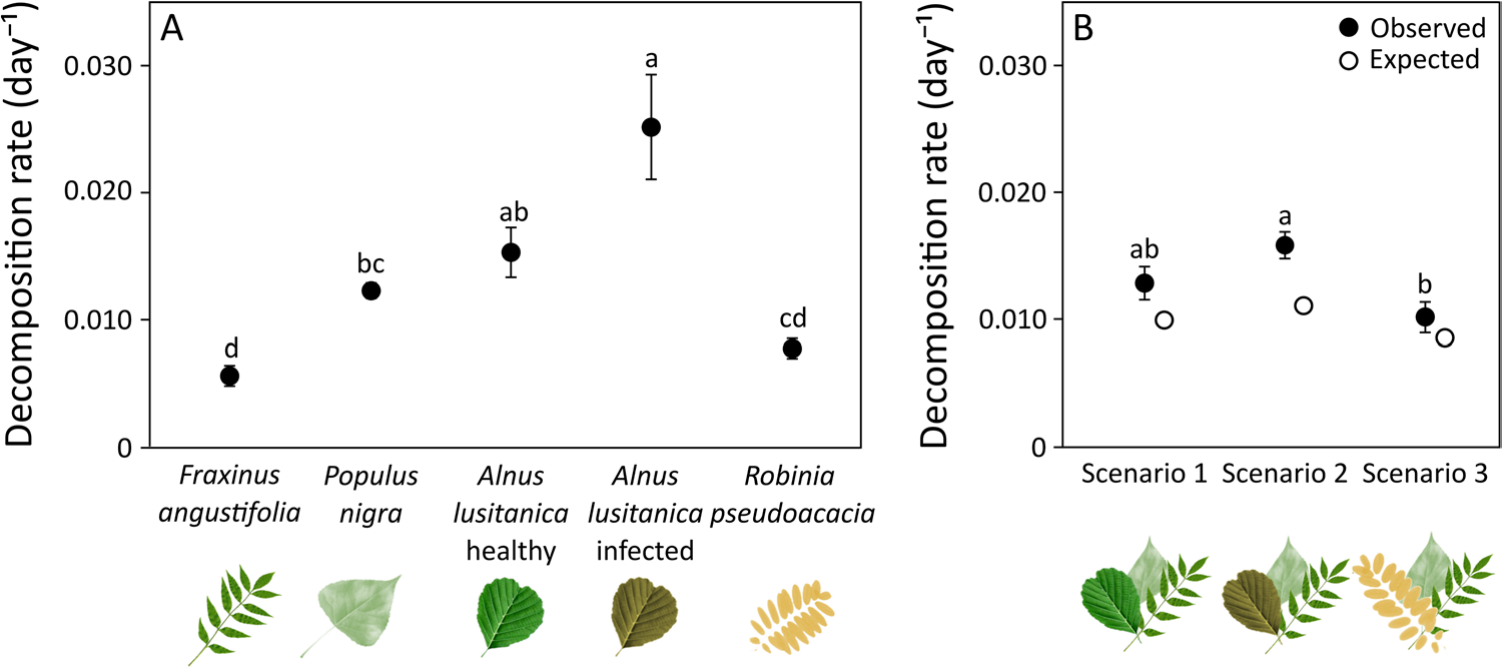
Decomposition rate (mean ± s.e.; *n* = 9) of leaf litter of five monocultures (**A**) and three mixtures (scenarios; **B**). Different letters indicate significant differences among leaf litter types or scenarios, analyzed with Tukey’s tests

Leaf litter types significantly differed in their decomposition rates (*p*-value < 0.001; Table S2, Fig. 1A), with (i) native species showing a gradient from lowest in ash to highest in alder; (ii) infected alder tending to decompose faster than healthy alder, with the difference being non-significant; (iii) and black locust decomposing more slowly than alder (Fig. 1A). Leaf litter decomposition varied with the scenario (*p*-value = 0.008), being significantly higher (56%) in scenario 2 than in scenario 3, 23% higher in scenario 2 than in scenario 1, and 21% lower in scenario 3 than in scenario 1, although the last two differences were non-significant (Table S2, Fig. 1B). All scenarios had higher decomposition rates than expected from monocultures (19–43%), with a positive net diversity effect in all of them (Fig. 1B). The net diversity effect increased with time (*p*-value = 0.001) and it was mainly driven by a positive complementarity effect, with a much lower and negative selection effect (Table S3). Diversity effects changed with scenario (net diversity effect, *p*-value = 0.014; complementarity effect, *p*-value = 0.007; selection effect, *p*-value = 0.010), with net diversity and complementarity effects being higher in scenario 2 than in scenario 3 (Table S3, Figure S1).

### Fungal Biomass

**Fig. 2:**
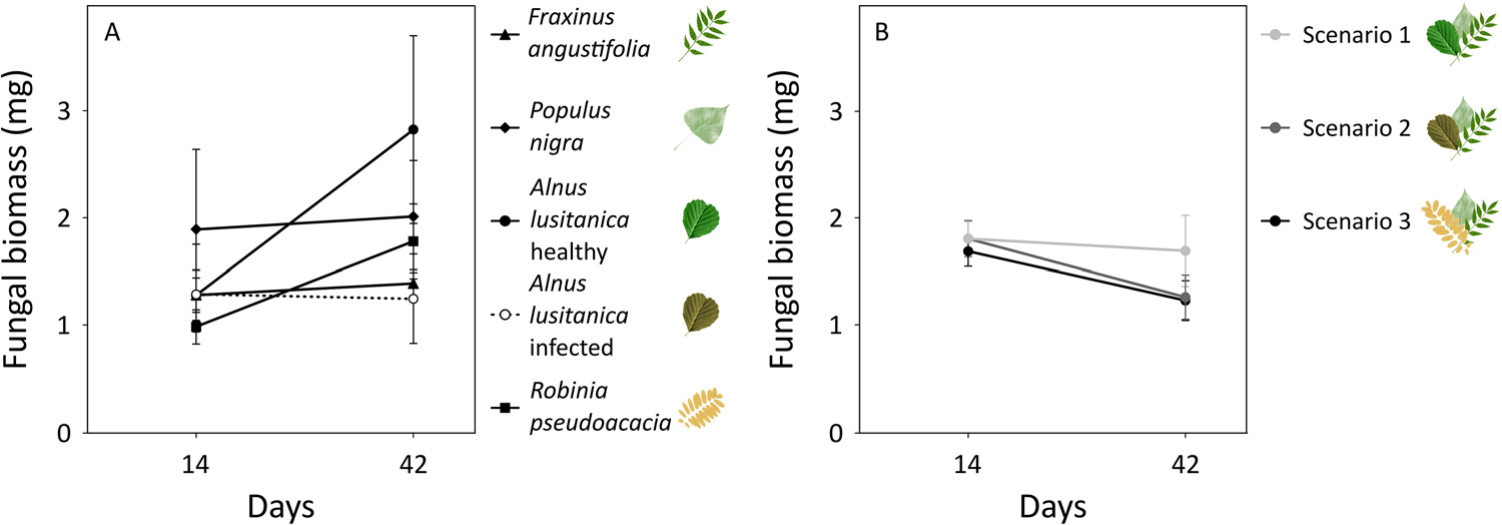
Fungal biomass (mean ± s.e.; *n* = 3) associated with leaf litter of monocultures (**A**) and mixtures (scenarios; **B**) at days 14 and 42

Fungal biomass was not significantly affected by leaf litter type (*p*-value = 0.422), although it tended to be higher in healthy alder and poplar at the later stages of the experiment (Table S3, Fig 2A); and it did not vary among scenarios (*p*-value = 0.384). However, fungal biomass decreased with time in all scenarios (*p*-value = 0.050), with a sharper decrease in scenarios 2 and 3 than in scenario 1 (Table S3, Fig. 2B), although it increased or did not change with time in monocultures. The net diversity effect on fungal biomass was mainly driven by a complementarity effect, with no differences among scenarios either for the net diversity effect (*p*-value = 0.280) or the complementary effect (*p*-value = 0.436), but a weak difference for the selection effect (*p*-value = 0.014) (Table S3, Fig. S2). The net diversity effect changed with time, from null or slightly positive at the first sampling date (10–16% higher than expected; Fig. S2A), to negative at the end of the experiment (Fig. S2D) (*p*-value < 0.001; Table S3). Despite the non-significant differences among scenarios at the end of the experiment, it was 41% lower than expected in scenario 3 and 9% lower than expected in scenario 1 (Fig. S2D). The net diversity effect was driven by a complementarity effect, which presented the same pattern, shifting with time from slightly positive (Fig. S2B) to negative values (Fig. S2E) (*p*-value < 0.001; Table S3). The selection effect was also affected by time (*p*-value = 0.032), remaining null in scenario 1 and negative in scenarios 2 and 3 (Table S3, Fig. S2C, Fig. S2F).

### Fungal Conidial Production and Assemblage Structure

**Fig. 3:**
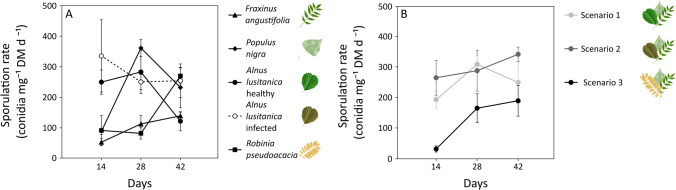
Fungal sporulation rates (mean ± s.e.; *n* = 3) associated with leaf litter of monocultures (**A**) and mixtures (scenarios; **B**) at days 14, 28, and 42

Sporulation rates varied with leaf litter type (*p*-value < 0.001), time (*p*-value < 0.001), and their interaction (*p*-value < 0.001) (Table S3); they were significantly higher in infected alder than in ash, poplar, and black locust, and lowest in ash (Fig. 3A). The scenarios also differed in sporulation rates (*p*-value = 0.001; Table S3), being higher in scenarios 1 and 2 than in scenario 3 (Fig. 3B). Species richness was affected by leaf litter type (*p*-value = 0.005), time (*p*-value = 0.038), and their interaction (*p*-value = 0.008) (Table S3), being lower at day 42 than at day 28. However, differences among leaf litter types were obscured by the interaction with time. Species richness was also affected by the scenario (*p*-value = 0.017; Table S3), but post hoc tests did not identify differences, only a trend of higher richness in scenario 2 (11 species) compared to scenarios 1 and 3 (9 species in each) (Table 2).

**Table 2:** Specific fungal sporulation rates (mean across sampling dates; *n* = 3) in leaf litter of five monocultures and three mixtures (scenarios). Accumulated species richness is given at the bottom of the table. Scenario 1: *Fraxinus angustifolia* + *Populus nigra* + *Alnus lusitanica* healthy; scenario 2: *Fraxinus angustifolia* + *Populus nigra* + *Alnus lusitanica* infected; scenario 3: *Fraxinus angustifolia* + *Populus nigra* + *Robinia pseudoacacia*

Fungal species	*Fraxinus angustifolia*	*Populus nigra*	*Alnus lusitanica* healthy	*Alnus lusitanica* infected	*Robinia pseudoacacia*	Scenario 1	Scenario 2	Scenario 3
*Alatospora acuminata* Ingold	3.35	24.67	26.76	44.81	6.66	10.08	13.77	5.98
*Alatospora pulchella* Marvanová			0.25	1.27	0.28		0.28	
*Anguillospora filiformis* Greath.	10.96	3.89	2.90	3.61	0.69	4.54	9.14	4.11
*Articulospora tetracladia* Ingold	25.77	149.64	141.99	121.37	47.12	147.36	125.31	67.70
*Flagellospora curvula* Ingold	0.62	0.84	1.39	5.28	2.08	2.52	3.49	0.55
*Hydrocina chaetocladia* Scheuer	0.12	0.91	0.30	0.59	0.21	2.40	3.20	0.32
*Lunuslospora curvula* Ingold	0.20	6.11	5.20	2.71	0.40	13.78	28.27	4.94
*Neonectria lugdunensis* (Sacc. & Therry) L. Lombard & Crous	0.03				0.31			
*Stenocladiella neglecta* (Marvanová & Descals) Marvanová & Descals	0.11	0.22		0.73		1.26	0.95	0.10
*Taeniospora gracilis* Marvanová			0.10	0.48			0.07	
*Tetrachaetum elegans* Ingold	49.92	31.85	16.80	7.05	6.11	23.16	47.57	24.85
*Triscelophorus acuminatus* Nawawi	11.13	9.60	22.93	92.60	84.36	45.49	66.34	20.29
Accumulated species richness	10	9	10	11	10	9	11	9

**Fig. 4:**
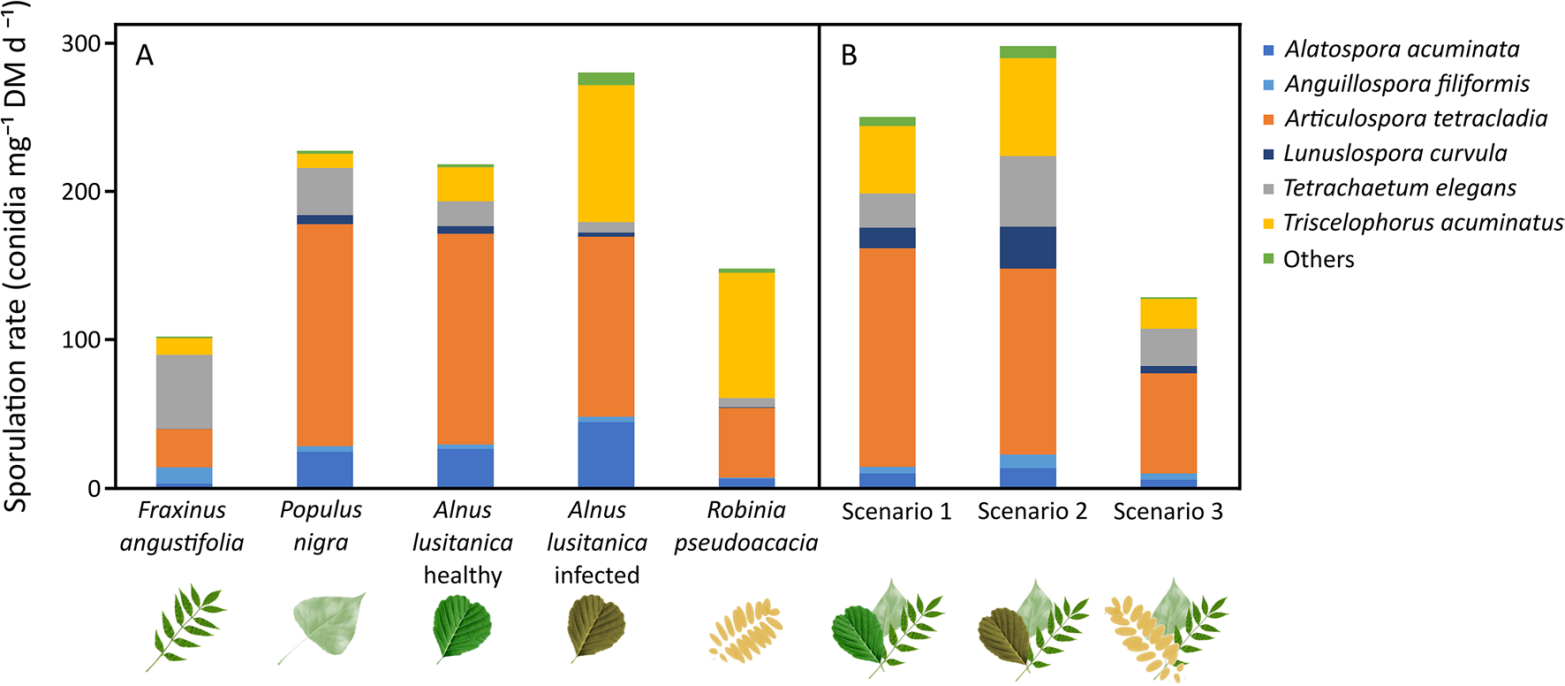
Fungal sporulation rates (average across sampling dates; *n* = 3) associated with leaf litter of monocultures (**A**) and mixtures (scenarios; **B**). “Others” include species contributing less than 5% to total conidial production

Fungal assemblage structure varied depending on the leaf litter type, time, and their interaction (*p*-values < 0.001; Table S4). Each leaf litter type showed a different response to time and assemblages in the third sampling date significantly differed from those in the first and second dates; e.g., *Triscelophorus acuminatus* Nawawi increased (*p*-value < 0.010). Despite variations due to the interaction with time, post hoc tests showed significant differences among all leaf litter types, except between healthy and infected alder and between healthy alder and poplar. Assemblages in poplar and healthy and infected alder leaf litter were dominated by *Articulospora tetracladia* Ingold (*p*-value < 0.001), followed by *Alatospora acuminata* Ingold (*p*-value = 0.003), and differed by the high relative contribution of *Tetrachaetum elegans* Ingold (*p*-value = 0.007) in poplar, the high relative contribution of *Lunulospora curvula* Ingold (*p*-value = 0.021) in healthy alder, and a high relative contribution of *T. acuminatus* (*p*-value = 0.005) in infected alder. Infected alder was also characterized by the occurrence of *Flagellospora curvula* Ingold (*p*-value = 0.006) and *Alatospora pulchella* Marvanová (*p*-value = 0.013), two to five times more abundant than in the other leaf litter types (Table 2). In black locust, *T. acuminatus* (*p*-value = 0.005) was also dominant. Ash had lower total sporulation, included *T. elegans* as the dominant species (*p*-value = 0.007), and showed high abundance of *Anguillospora filiformis* Greath (*p*-value < 0.001) and *A. tetracladia* (Fig. 4A, Table 2).

Assemblage structure also varied with scenario, time, and their interaction (*p*-values ≤ 0.002; Table S4). Assemblages in the last sampling date differed significantly from those in the first and second dates and were characterized by a higher proportion of *T. acuminatus* (*p*-value < 0.001). Assemblages in scenarios 1 and 2 differed from those in scenario 3 (Table S4), because total sporulation was higher and *A. tetracladia*, the dominant species, also presented a higher sporulation in scenarios 1 and 2 (*p*-value = 0.046), with *A. filiformis* showing a higher relative contribution in scenario 3 (*p*-value = 0.046) (Fig. 4B, Table 2).

## Discussion

Our results revealed changes in stream ecosystem functioning and associated fungal assemblages following infection of riparian alder by *P.* ×*alni* and subsequent replacement of diseased trees by the invasive black locust. Rates of leaf litter decomposition, a fundamental process often used to assess ecological impacts of stressors on ecosystem functioning [44, 45], experienced first a weak increase after alder infection and subsequently a strong decrease after replacement of infected alder with black locust, when measured in leaf litter mixtures also containing other native species (i.e., ash and poplar). These changes had the expected direction, confirmed hypotheses i and ii, and reflected differences in leaf litter chemistry. Leaf litter of infected alder showed higher nutrient concentrations and lability than that of healthy alder, as reported before [24], which has been attributed to reduced nutrient resorption efficiency of infected trees [46] or to accumulation of nutrients in leaves due to stress induced by root damage [47]. In contrast, black locust leaf litter had lower N concentration than that of alder and the lowest P concentration, as well as high polyphenol concentrations, which reduced its palatability and hence its decomposition rate, as shown also in other studies [33–35, 48].

Despite the clear differences in leaf litter chemistry and the fact that trends in leaf litter decomposition followed the expected pattern, the reduction in decomposition rates when comparing mixtures with healthy alder (scenario 1) with mixtures with black locust (scenario 3) was not statistically significant during our experiment, which lasted 42 days. In contrast, fungal sporulation rates were significantly lower in mixtures with black locust than in those with healthy alder, which was likely caused by the high concentration of polyphenols in black locust, which are known to have anti-microbial activity [12, 34, 49, 50]. The lower reproduction rate of microbial decomposers in mixtures with black locust suggests that decomposition rates might be significantly reduced if measured further in the longer term. This is supported by the reduction in fungal biomass that occurred with time in mixtures with black locust, but not in mixtures with healthy alder. It should be considered that the disappearance of alder generally occurs after a period when healthy alder is infected. Thus, while decomposition and fungal sporulation rates of infected alder leaf litter were slightly higher than those of healthy alder leaf litter (in monocultures and in scenario 1 vs. 2), this increase was large enough to obtain a significant reduction in decomposition and sporulation rates when infected trees (scenario 2) were further replaced by black locust (scenario 3).

All leaf litter mixtures showed a positive net diversity effect on decomposition, that is, they decomposed faster than expected based on the decomposition of individual species. The net diversity effect was mainly driven by a complementarity effect, as shown for the decomposition of other leaf litter mixtures [35, 51], and it increased with time, also as observed previously [52]. Alder leaf litter has been reported to enhance decomposition of more recalcitrant leaf litter in mixtures [12, 51, 53], either by attracting detritivores [54] or by increasing the leaf nutrient concentration of other species through nutrient transfer by fungal hyphae [55]. In our experiment, there could have been transfer of N from alder or black locust to the other species, and also a transfer of P in the opposite direction. This could possibly occur through uptake of leached nutrients [56, 57], since leaf discs were individually in constant movement in the microcosms, most likely precluding nutrient transfer by fungal hyphae.

In contrast to the pattern revealed for decomposition, we found no significant differences among leaf litter mixtures for fungal biomass, with only a weak trend towards higher biomass in healthy alder. This contrasts with previous studies, where infected alder showed higher fungal biomass than healthy alder [24]. Here, infected alder showed similar values to those of black locust, which usually presents low fungal biomass due to their low nutrient and high polyphenol concentrations [34]. Our observed pattern could be caused by nutrient transfer among leaf litter types, which is supported by the positive complementarity effect on fungal biomass found at the early stages of the experiment. Towards later stages, however, there was a negative complementarity effect, suggesting that inhibitory compounds present in different leaf litter types could have reduced fungal growth [53, 54], maybe in parallel to a depletion of nutrients [58]. The greater reduction in mixtures with black locust and infected alder may be caused by the higher polyphenol and lower nutrient concentration in black locust, which make them more prone to negative effects [34], and the enhancement of decomposition caused by infected alder, which may have caused a faster consumption of nutrients [58], which therefore may not have compensated the negative effects of tannins and other compounds [12].

Leaf litter of different species showed different fungal assemblages, according to the different substrate preferences shown by fungal species [11]. As previously observed [24], healthy and infected alder showed similar assemblages, but mixtures with alder had different assemblages compared to mixtures with black locust. This difference was mainly driven by *A. tetracladia*, which was the most abundant hyphomycete in all mixtures, but it was reduced in the presence of black locust. This probably occurred because its sporulation was limited due to lower nutrient and higher polyphenol concentrations [14, 54, 59, 60].

## Conclusions

Our study reveals changes in the key stream ecosystem process of leaf litter decomposition and associated fungal assemblages, following a sequence of alterations in species composition of the riparian forest. The most striking changes occur when alder is infected by *P.* ×*alni*, which slightly accelerates the process of leaf litter decomposition if compared to non-infected alder trees and which, after the replacement of alder with the exotic black locust, induces a significant reduction in the decomposition process and a substantial change in the characteristics of microbial decomposer assemblages. Our results also suggest that changes detected 42 days after the experiment started would be intensified in the longer term, as proposed elsewhere [24]. Impacts to the riparian forest, such as infectious diseases—which drive changes in the traits of native species and can ultimately lead to their loss—and species invasions—which introduce species with different traits from those of native species—have the capacity to alter the functioning and structure of the stream ecosystem. Our study shows how this occurs even if the native and invasive species belong to the same functional group, in this case N-fixing species. Overall, our study highlights the importance of preserving native and healthy riparian vegetation in order to maintain proper ecosystem functioning.

### Supplementary Information

ESM 1 (PDF 596 kb)

## Data Availability

Data is available from the Open Science Framework Repository.
