# Supplementary material for: Dieback and Replacement of Riparian Trees May Impact Stream Ecosystem Functioning
Source: Microb Ecol. 2024 Jan 16;87(1):32. doi: 10.1007/s00248-024-02343-w (PMC10791780; doi:10.1007/s00248-024-02343-w)

Dieback and replacement of riparian trees may impact stream ecosystem functioning

Microbial Ecology

Alberto Alonso[1]*, Luz Boyero[1,2], Alejandro Solla[3] and Verónica Ferreira[4]

[1]Department of Plant Biology and Ecology, Faculty of Science and Technology, University of the Basque Country (UPV/ EHU), Leioa, Spain

[2]Basque Foundation for Science, IKERBASQUE, Bilbao, Spain

[3]Faculty of Forestry, Institute for Dehesa Research (INDEHESA), University of Extremadura, Avenida Virgen del Puerto 2, 10600, Plasencia, Spain

[4]MARE – Marine and Environmental Sciences Centre, ARNET – Aquatic Research Network, Department of Life Sciences, University of Coimbra, Calçada Martim de Freitas, 3000-456 Coimbra, Portugal

*Corresponding author: alberto.alonso@ehu.eus

Table S1. Results of ANOVA exploring the effects of leaf litter types (*Fraxinus angustifolia, Populus nigra, Alnus lusitanica* healthy, *A. lusitanica* infected and *Robinia pseudoacacia*) on leaf litter physico-chemical traits: C (carbon), N (nitrogen), P (phosphorus), polyphenol and lignin concentrations (% dry mass), elemental ratios (C:N, C:P and N:P) and leaf toughness (kPa). df = degrees of freedom, F = F-statistic, p = p-value.

Variable	df	F	p
C	4, 10	189.10	<0.001
N	4, 10	271.32	<0.001
P	4, 10	72.87	<0.001
Polyphenol	4, 10	7.19	0.005
Lignin	4, 10	610.02	<0.001
C:N	4, 10	254.25	<0.001
C:P	4, 10	36.43	<0.001
N:P	4, 10	30.21	<0.001
Toughness	4, 10	20.41	<0.001

Table S2. Results of ANCOVA exploring the effects of leaf litter types (*Fraxinus angustifolia*, *Populus nigra*, *Alnus lusitanica* healthy, *A. lusitanica* infected and *Robinia pseudoacacia*) and scenarios (scenarios 1, 2 and 3), with time as a covariate, on fraction leaf mass remaining. df = degrees of freedom, F = F-statistic, p = p-value.

**Treatment**	**Factor**	**df**	**F**	**p**
Species	Species	4	14.57	<0.001
	Time	2	11.84	<0.001
Scenario	Scenario	1	5.99	0.008
	Time	2	21.82	<0.001

Table S3. Results of ANOVA exploring the effects of leaf litter types (*Fraxinus angustifolia*, *Populus nigra*, *Alnus lusitanica* healthy, *A. lusitanica* infected and *Robinia pseudoacacia*) or scenarios (scenarios 1, 2 and 3), time and their interaction, on net diversity effect, complementarity effect and selection effect on leaf litter mass loss, sporulation rate (number of conidia mg^{-1} DM d^{-1}), species richness (number of species $sample^{-1}$), fungal biomass (mg fungal DM) and net diversity effect, complementarity effect and selection effect on fungal biomass. df = degrees of freedom, F = F-statistic, p = p-value.

**Variable**	**Treatment**	**Factor**	**df**	**F**	**p**
Net diversity effect on leaf mass loss	Scenario	Scenario	2, 18	5.41	0.014
		Time	2, 18	12.03	0.001
		Scenario×Time	4, 18	0.23	0.920
Complementarity effect on leaf mass loss	Scenario	Scenario	2, 18	6.55	0.007
		Time	2, 18	13.67	<0.001
		Scenario×Time	4, 18	0.16	0.958
Selection effect on leaf mass loss	Scenario	Scenario	2, 18	5.97	0.010
		Time	2, 18	25.85	<0.001
		Scenario×Time	4, 18	1.13	0.372
Fungal biomass	Species	Species	4, 20	1.02	0.422
		Time	1, 20	3.21	0.088
		Species×Time	4, 20	1.27	0.317
	Scenario	Scenario	2, 12	1.04	0.384
		Time	1, 12	4.76	0.050
		Scenario×Time	2, 12	0.60	0.566
Net diversity effect on fungal biomass	Scenario	Scenario	2, 12	1.42	0.280
		Time	1, 12	41.74	<0.001
		Scenario×Time	2, 12	0.79	0.476
Complementarity effect on fungal biomass	Scenario	Scenario	2, 12	0.89	0.436
		Time	1, 12	51.91	<0.001
		Scenario×Time	2, 12	0.81	0.467
Selection effect on fungal biomass	Scenario	Scenario	2, 12	6.22	0.014
		Time	1, 12	5.87	0.032
		Scenario×Time	2, 12	1.14	0.353
Sporulation rate	Species	Species	4, 30	10.83	<0.001
		Time	2, 30	10.01	<0.001
		Species×Time	8, 30	4.86	<0.001
	Scenario	Scenario	2, 18	10.53	0.001
		Time	2, 18	4.07	0.035
		Scenario×Time	4, 18	0.76	0.566
Species richness	Species	Species	4, 30	4.62	0.005
		Time	2, 30	3.67	0.038
		Species×Time	8, 30	3.29	0.008
	Scenario	Scenario	2, 18	5.18	0.017
		Time	2, 18	0.93	0.414
		Scenario×Time	4, 18	1.20	0.343

Table S4. Results of PERMANOVA exploring the effects of leaf litter types (*Fraxinus angustifolia*, *Populus nigra*, *Alnus lusitanica* healthy, *A. lusitanica* infected and *Robinia pseudoacacia*) and scenarios (scenarios 1, 2 and 3), time and their interaction on fungal assemblages. df = degrees of freedom, F = F-statistic, R^2 = R squared, p = p-value.

**Treatment**	**Factor**	**df**	**F**	**R^2**	**p**
Species	Species	4	9.13	0.36	<0.001
	Time	2	5.73	0.11	<0.001
	Species×Time	8	2.74	0.22	<0.001
Scenario	Scenario	2	4.89	0.19	<0.001
	Time	2	6.05	0.23	<0.001
	Scenario×Time	4	2.91	0.23	0.002

Fig. S1. Net diversity (A), complementarity (B) and selection (C) effects on leaf litter decomposition (measured as fraction of litter mass loss) in scenarios 1, 2 and 3 using the mean of the three sampling dates. Symbols are means, whiskers are upper and lower bounds of 95% nonparametric bootstrapped confidence intervals and different letters indicate significant differences among scenarios.

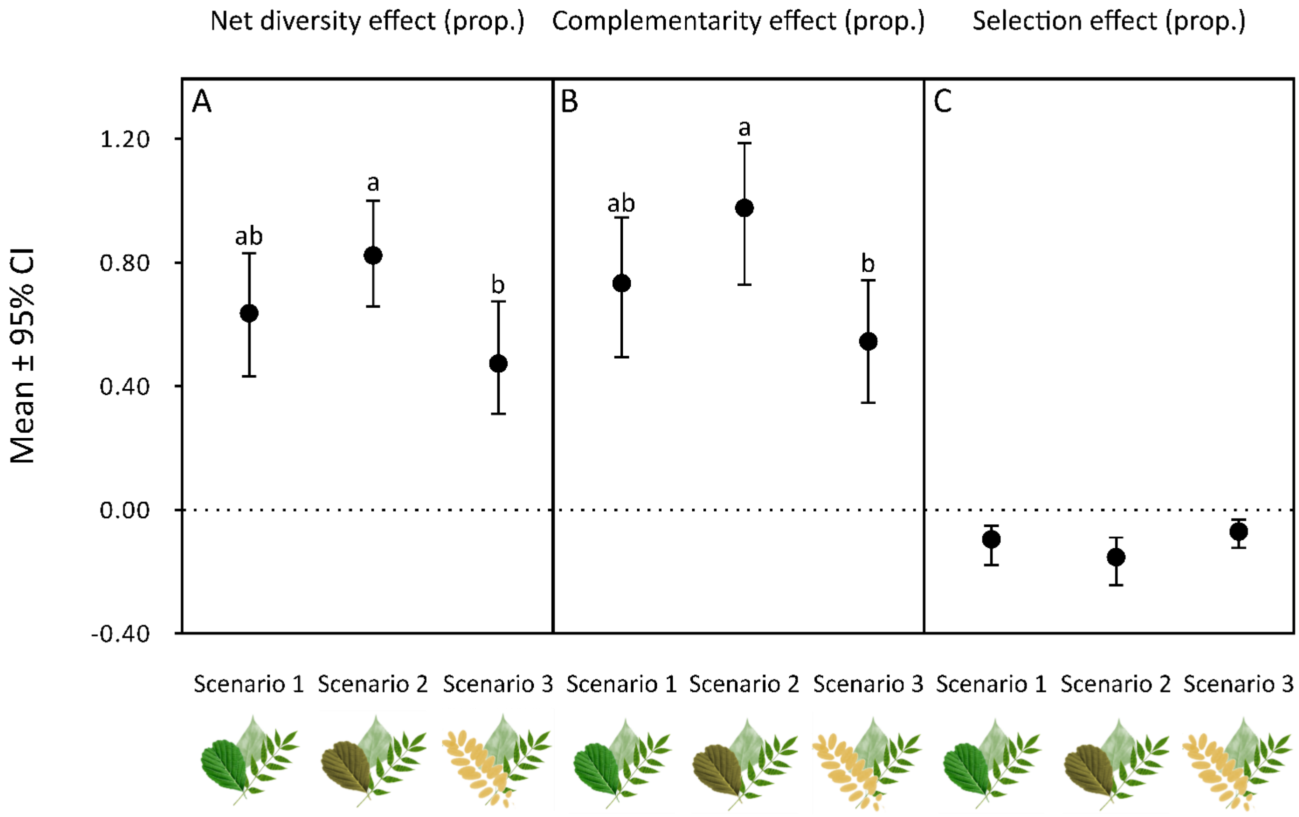

Fig. S2. Net diversity (A, D), complementarity (B, E) and selection (C, F) effects on fungal biomass (mg) in scenarios 1, 2 and 3 at days 14 and 42. Symbols are means, whiskers are upper and lower bounds of 95% nonparametric bootstrapped confidence intervals.

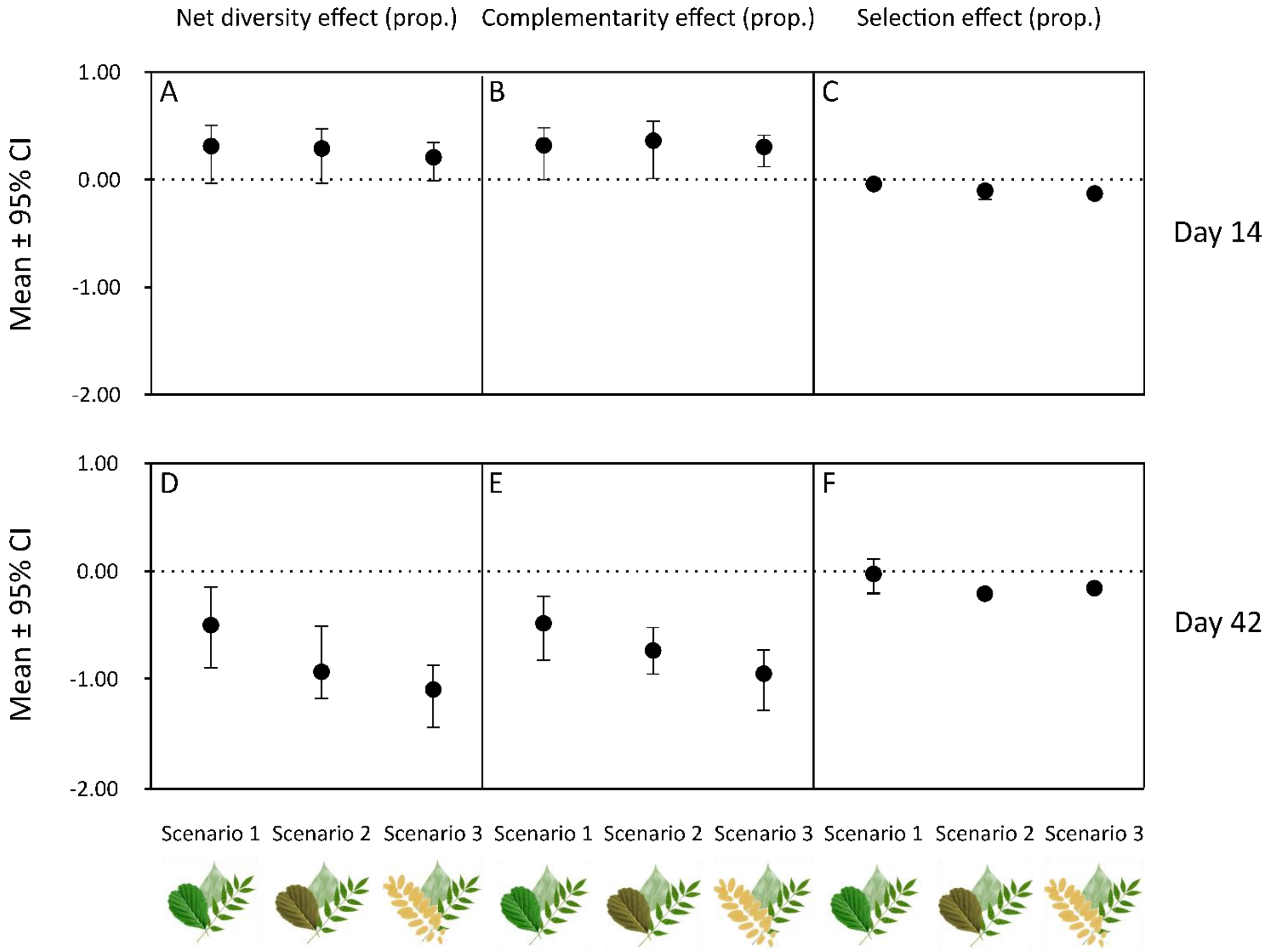

Supplement: Supplementary file 1 — (PDF 596 kb) [file 248_2024_2343_MOESM1_ESM.pdf]
